# Successful troubleshooting for “stone-basket impaction” using argon plasma coagulation and a novel device delivery system

**DOI:** 10.1055/a-2134-7127

**Published:** 2023-08-21

**Authors:** Koichiro Mandai, Hiroaki Satake, Takato Inoue

**Affiliations:** Department of Gastroenterology, Kyoto Second Red Cross Hospital, Kyoto, Japan

Various techniques, including the use of a transoral endotripter, extracorporeal shock wave lithotripsy, and electrohydraulic lithotripsy (EHL), have been reported to resolve basket catheter impaction 1 . Additionally, argon plasma coagulation (APC) may be effective in cutting metal wires 2 . We describe a successful troubleshooting method to resolve stone-basket impaction that is achieved by cutting the basket wires using APC and removing them via a novel device delivery system.

An 88-year-old man was referred to our hospital with obstructive jaundice caused by a biliary stone. Endoscopic retrograde cholangiopancreatography (ERCP) showed a 12-mm stone in the common bile duct. A mechanical lithotripter (StoneSmash; Boston Scientific, Massachusetts, USA) was used in an attempt to remove the stone, but the stone could not be crushed and the lithotripter became stuck in the bile duct. Rescue therapy using a transoral endotripter failed and emergent EHL was not available. After biliary stenting had been performed, the wires were cut in the duodenum, taking 5 minutes, with APC (VIO300 D with APC2; 80 W, flow rate 2 L/min; ERBE Elektromedizin, Tübingen, Germany) ( Fig. 1 a ). Thereafter, it was possible to extract the body of the mechanical lithotripter ( Fig. 1 b ), but the impacted wires and entrapped stone remained in the bile duct.

**Fig. 1:**
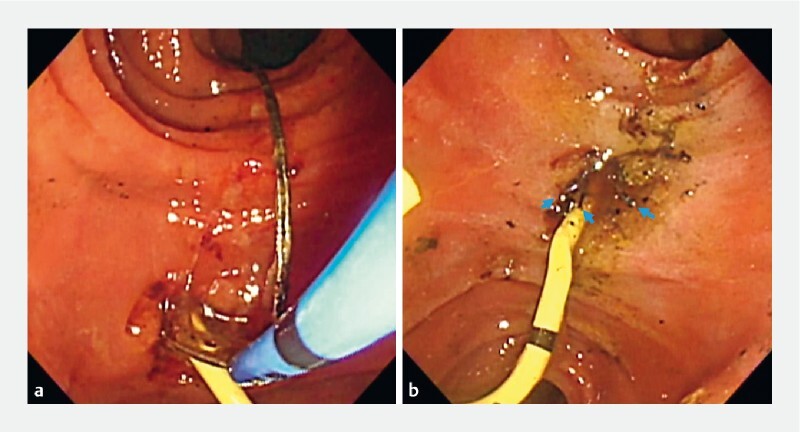
Endoscopic images showing: **a** the basket wire about to be cut by argon plasma coagulation in the duodenum; **b** the extracted body of the mechanical lithotripter and the cut wire ends exposed to the duodenum (blue arrows).

A second ERCP was performed 1 week later to remove these. The cut wire ends had migrated into the common bile duct ( Fig. 2 a ). After the stent had been removed, an endoscopic sheath (outer and inner diameters 2.4 mm and 2.06 mm, respectively; Endosheather; Piolax Medical Devices, Kanagawa, Japan) 3 was inserted over the guidewire. The inner catheter and guidewire were removed, leaving the outer sheath in place. A biopsy forceps (diameter 1.8 mm; Radial Jaw; Boston Scientific) was inserted through the outer sheath, and the migrated wires were successfully grasped with the forceps and removed ( Fig. 2 b ). The stones were then crushed by cholangioscopy-guided EHL and removed using a balloon catheter ( Video 1 ).

**Fig. 2:**
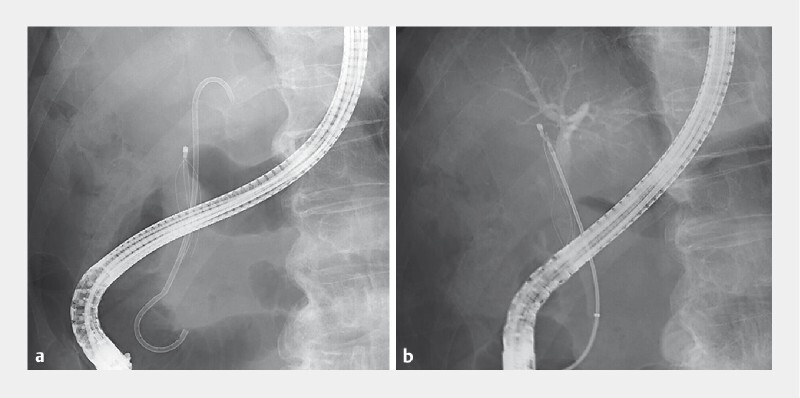
Fluoroscopic images showing: **a** the cut wire ends that had migrated into the common bile duct; **b** the migrated wires grasped with a forceps that had been inserted through a novel delivery system.

**Video 1** An impacted basket wire catheter is cut by argon plasma coagulation and the migrated wires within the common bile duct are removed with forceps inserted through a novel device delivery system, allowing cholangioscopy-guided electrohydraulic lithotripsy to be performed, with subsequent successful removal of the stones.


Endoscopy_UCTN_Code_CPL_1AK_2AF

## Notes

Endoscopy E-Videos

https://eref.thieme.de/e-videos
*E-Videos* is an open access online section of the journal *Endoscopy* , reporting on interesting cases and new techniques in gastroenterological endoscopy. All papers include a high-quality video and are published with a Creative Commons CC-BY license. Endoscopy E-Videos qualify for HINARI discounts and waivers and eligibility is automatically checked during the submission process. We grant 100% waivers to articles whose corresponding authors are based in Group A countries and 50% waivers to those who are based in Group B countries as classified by Research4Life (see: https://www.research4life.org/access/eligibility/ ). This section has its own submission website at https://mc.manuscriptcentral.com/e-videos
